# Identification of the Flavone-Inducible Counter-Defense Genes and Their *cis*-Elements in *Helicoverpa armigera*

**DOI:** 10.3390/toxins15060365

**Published:** 2023-05-29

**Authors:** Zhongyuan Deng, Yuting Zhang, Liying Fang, Min Zhang, Lixiang Wang, Xinzhi Ni, Xianchun Li

**Affiliations:** 1 School of Agricultural Sciences, Zhengzhou University, Zhengzhou 450001, China; dzy@zzu.edu.cn (Z.D.); zhyuting2021@163.com (Y.Z.); fangliyingaa@163.com (L.F.); zhangmin753@gmail.com (M.Z.); wanglixiang@zzu.edu.cn (L.W.); 2 School of Life Sciences, Zhengzhou University, Zhengzhou 450001, China; 3 USDA-ARS, Crop Genetics and Breeding Research Unit, University of Georgia-Tifton Campus, Tifton, GA 31793, USA; xinzhi.ni@usda.gov; 4 Department of Entomology and BIO5 Institute, University of Arizona, Tucson, AZ 85721, USA

**Keywords:** *Helicoverpa armigera*, flavone, detoxification metabolism, carboxylesterase, *cis*-transcriptional regulation, ARE

## Abstract

Flavone is widely found in plants and plays an important role in plant defense against pests. Many pests, such as *Helicoverpa armigera*, use flavone as a cue to upregulate counter-defense genes for detoxification of flavone. Yet the spectrum of the flavone-inducible genes and their linked *cis*-regulatory elements remains unclear. In this study, 48 differentially expressed genes (DEGs) were found by RNA-seq. These DEGs were mainly concentrated in the retinol metabolism and drug metabolism-cytochrome P450 pathways. Further in silico analysis of the promoter regions of 24 upregulated genes predicted two motifs through MEME and five previously characterized *cis*-elements including CRE, TRE, EcRE, XRE-AhR and ARE. Functional analysis of the two predicted motifs and two different versions of ARE (named ARE1 and ARE2) in the promoter region of the flavone-inducible carboxylesterase gene *CCE001j* verified that the two motifs and ARE2 are not responsible for flavone induction of *H. armigera* counter-defense genes, whereas ARE1 is a new xenobiotic response element to flavone (XRE-Fla) and plays a decisive role in flavone induction of *CCE001j*. This study is of great significance for further understanding the antagonistic interaction between plants and herbivorous insects.

## 1. Introduction

Chemical pesticides have been the most effective tools for control of agricultural pests, but overuse or misuse of them may lead to environmental pollution and pesticide residues in water, soil, and crops, which eventually harm human health [1]. In addition, the abuse of pesticides generally causes the evolution of resistance by pests [2]; the harmful effects of pesticides on the environment can endanger the sustainability of ecosystems [3]. Therefore, it is very important to find eco-friendly biopesticides with target ability, degradability, and low toxicity. Secondary metabolites such as alkaloids, flavonoids, terpenoids, and sterols extracted from plants are important resources [4]. These biopesticides usually degrade quickly and therefore do not last long in the ecosystem [5]. Studies on the effects of these compounds on insect pests provide new opportunities for the development of new biological insecticides.

During the long evolutionary process, plants have developed defense mechanisms against insect feeding through morphological, biochemical, and molecular regulation [6]. An important defense strategy of plants against insects is to change the content of secondary metabolites [7]. Plants acquire the ability to produce large amounts of secondary metabolites through accidental mutations and gene recombination [8]. Secondary metabolites, also known as allelochemicals, are not produced by the normal physiological reactions of plants but are produced as major metabolic by-products [9,10]. Allelochemicals can act as insect repellents, deterrents, anti-nutrients, and anti-digestion compounds to reduce the development and survival of herbivorous insects [8].

Plants possess an allelochemical-based chemical defense system. Insects have evolved a variety of counter-defense strategies against allelochemicals, including behavioral avoidance, rapid excretion, enhanced degradation, and target site insensitivity [11]. It has been well known that detoxifying enzyme systems in insects plays a key role in dealing with plant secondary metabolites [12]. Studies have shown that molecular adaptation makes enzymes related to digestion, protection, and detoxification more active in insects. This involves the rapid synthesis of hydrolases, antioxidant enzyme, and CYP450s, a physiological response to foreign chemicals that is essential for insect survival and reproduction [13]. Upon exposure to the allelochemicals of host plants, insects upregulate their counter-defense level, which helps them to survive in a toxic environment.

Flavonoids are widely found in plants and represent a group of allelochemicals with biological and chemical diversity [14]. Flavonoids protect plants from biological and abiotic stresses, including ultraviolet radiation, pathogen infections, and herbivores [14]. Like other groups of allelochemicals, flavonoids play an important role in plant defense against pests [15]. Flavonoids can reduce the survival and growth of *Ostrinia nubilalis* [16] and decrease the body weight and development time of *Mamestra configurata* larvae and pupae [17]. Flavonoids found in ginkgo biloba leaves have a negative anti-feeding effect on *Hyphantria cunea* larvae [18]. Quercetin, a flavonoid allelochemical, reduces the larval body weight of *Spodoptera litura* [10].

Insects rely on their detoxification enzymes, such as carboxylesterase (CarE), glutathione S-transferase (GST), and cytochrome P450s, to metabolize and detoxify these flavonoid allelochemicals [19]. For example, the honeybee P450 CYP6AS can metabolize the flavonoid quercetin [20]. In *Helicoverpa zea*, at least two specific P450s, CYP6B8 and CYP321A1, are associated with detoxification of flavone [21], another flavonoid. Furthermore, insects can also use allelochemicals as signals to enhance the expression and activity of their allelochemical-detoxifying enzymes [22,23,24]. For example, total P450s enzyme activity increased when *H. armigera* fed on host plants containing a large amount of gossypol [25]. Exposure to zanthoxylum toxin induced increased *CYP6B1* expression in the midgut and adipose body of *Plutella xylostella* [26]. Likewise, total CarE activity was significantly induced by the exposure of *Lymantria dispar* to phenolic glycosides [27]. *Sitobion avenae* fed on higher indole alkaloids showed higher CarE activity [28]. Such an allelochemical-triggered upregulation of detoxification genes is realized by the interaction between *cis*-regulatory elements and the corresponding *trans*-acting factors [24,29].

The flavonoid flavone is widely distributed in plants and plays an important role in plant defense against insect pests. It has been reported that insect pests such as *H. armigera* can counteract flavone by enhancing expression of flavone-detoxifying cytochrome P450 gene such as *CYP321A1* [30]. However, it is not clear what other genes and regulatory elements are involved in metabolism of flavone. In this study, we will reveal the flavone response genes by RNA-seq and identify the corresponding flavone-responsible *cis*-regulatory elements in the promoter regions of these genes in *H. armigera*. It is of great significance to further understand the antagonism between plants and herbivorous insects and to reveal the host adaptation mechanism of insects.

## 2. Results

### 2.1. Selection of a Flavone Dose for Induction of the Larval Midgut Transcriptome of H. armigera

**Figure 1:**
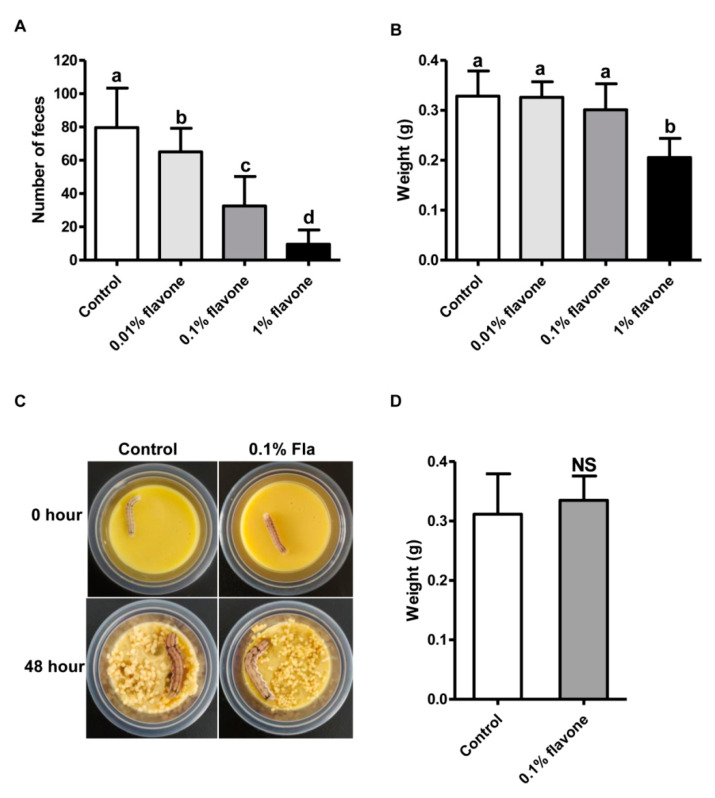
Flavone induction of *H. armigera* larvae: (**A**) Fecal quantity of the 5th instar larvae fed with 0.01%, 0.1%, and 1% flavone diets for 3 days. (**B**) Body weight gain of the 5th instar larvae fed with 0.01%, 0.1%, and 1% flavone diets for 3 days. (**C**) Photos of the 6th instar larvae before (0 h) and 48 h post feeding with 0.1% flavone diets. (**D**) Body weight gain of the 6th instar larvae fed with 0.1% flavone diets for 48 h. Bars with different letters such as a, b, c, d (*p* < 0.05, one-way ANOVA followed by Tukey’s HSD test) are significantly different. NS means no significantly different (*p* > 0.05, independent *t* test).

A bioassay of 5th instar larvae of the *H. armigera* Baiyun strain with diets incorporated with 0.01%, 0.1%, or 1% flavone was conducted to select a suitable induction dose that can affect larval midgut transcriptome but has no noticeable impacts on the growth of the larvae. Relative to the control larvae fed on normal diets, the larvae fed on any of the three flavone-containing diets excreted a lower amount of feces (Figure 1A). The higher the flavone concentration, the lower the fecal volume (Figure 1A). In terms of body weight, a measurement of larval growth, no significant differences were found between the control larvae and the larvae fed on 0.01% or 0.1% flavone diets (Figure 1B). However, the body weight of the larvae fed on 1% flavone diets was significantly reduced (Figure 1B), suggesting that 0.1% flavone is probably the suitable dose for induction of the larval transcriptome. A bioassay of newly-molted 6th instar larvae with 0.1% flavone diets further confirmed that 0.1% flavone is the suitable induction dose because no significant differences in morphology and body weight were detected between the experimental group and the control group (Figure 1C,D).

### 2.2. Sequences Reads Obtained from Flavone-Induced Larval Midgut Transcriptome

RNA sequencing (RNA-seq) of midgut RNA samples extracted from 48 h old 6th instar larvae of *H. armigera* fed with control (three biological replicates, hereafter named CK-1, CK-2, CK-3) or 0.1% flavone (three biological replicates, hereafter named Fla-1, Fla-2, and Fla-3) diets yielded a total of 37–60 million raw sequence reads per replicate (Table 1). The quality of these raw reads was very good, as more than 97% and 93% of bases had a quality score of ≥20 (Q20, 99% base call accuracy) and ≥30 (Q30, 99.9% base call accuracy), respectively (Table 1). After a series of cleaning steps, such as trimming adaptor sequence and low-quality bases and filtering low-quality reads, a total of 58,567,088 (99.80%, Fla-1), 47,994,546 (99.83%, Fla-2), 37,504,354 (99.91%, Fla-3), 37,459,394 (99.87%, CK-1), 60,101, 208 (99.84%, CK-2), and 52,184,106 (99.82%, CK-3) clean reads were obtained for each of the three RNA samples from the control and flavone treatment groups (Table 1). About 70–75% of the clean reads of the six RNA samples could be mapped to the genome of *H. armigera* (NCBI genome accession number: GCA_023701775.1). The GC content of the clean reads was similar (~55%) within and between the two groups (Table 1).

### 2.3. Analysis of Flavone-Elicited Differentially Expressed Genes

**Figure 2:**
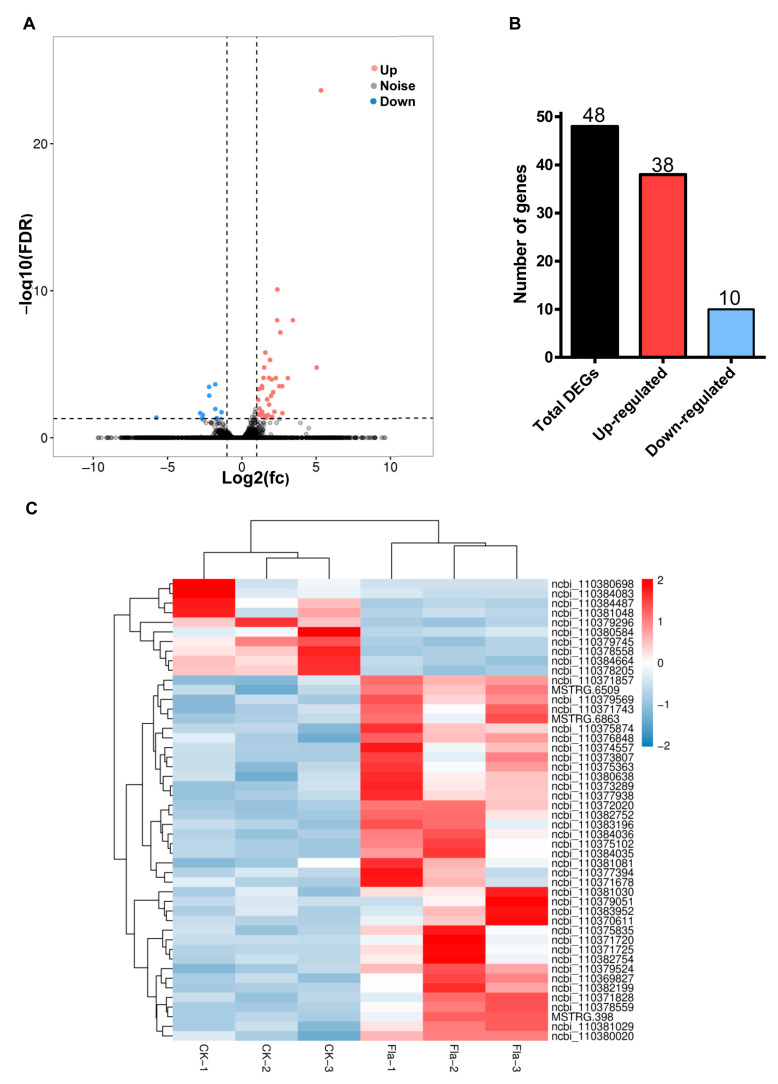
Statistical identification of up- and downregulated larval midgut genes after flavone induction: (**A**) Volcano plot between log_10_ significance (FDR, *Y*-axis) vs. log_2_ fold change (FC, *X*-axis). Red data points indicate upregulated genes, and blue data points indicate downregulated genes. (**B**) Numbers of up- and downregulated genes. (**C**) Heat map of differentially expressed genes (DEGs). *X*-axis: RNA sample name; *Y*-axis: DEGs. The colors indicate the log_2_ FC of DEGs.

Statistical analysis of the FPKM (reads per kilo base of exon model per million mapped reads) value of each gene expressed in the midgut of the control and flavone-treated larvae using DESeq2 uncovered 48 genes meeting the criteria (False discovery rate (FDR) < 0.05 and |log_2_ fold change (FC)| > 1) for differentially expressed genes (DEGs) (Figure 2A). Among the 48 DGEs, 38 and 10 genes were up- and downregulated (Figure 2B), respectively, by 0.1% flavone. Two-way hierarchical clustering analysis divided the six larval midgut RNA samples into control and flavone clades and the 48 DGEs into up- and downregulated clades (Figure 2C), confirming the RNA-seq data were reliable.

**Figure 3:**
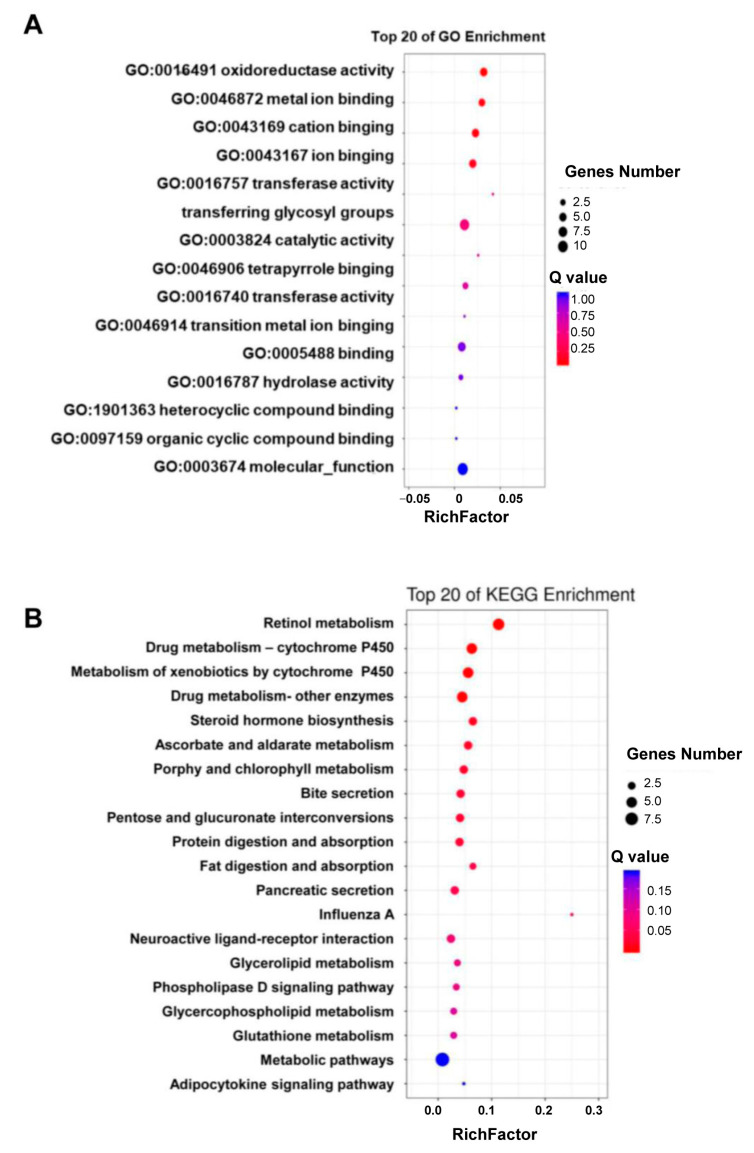
The enriched GO terms (**A**) and KEGG pathways (**B**) of the 48 DGEs.

Except for 4 DGEs that encoded synaptic vesicle 2-related protein, cytochrome b5, cuticle protein 4, or hemolin, the rest of the 48 DGEs encoded various enzymes that played a role in detoxification (nine P450s, six carboxylesterases, three UGTs, two GSTs, one epoxide hydrolase, and one alcohol dehydrogenase), digestion (seven proteases), lipid metabolism (two acyltransferase, one neutral lipase, one fatty alcohol acetyltransferase, and one androgen-induced gene 1 protein), ecdysone homeostasis (three ecdysteroid 22-kinases, one 3-dehydroecdysone 3alpha-reductase), retinol metabolism (two retinol dehydrogenases) or cuticle sclerotization (laccase) (Figure 2C). Consistent with their functional annotations and categories, the 48 DGEs were mainly enriched in the Gene Ontology (GO) terms of “oxidoreductase activity (GO:0016491”, ”metal ion binding (GO:0046872)”, and “cation binding (GO:0043169)” (Figure 3A) and the KEGG pathways of retinol metabolism, drug metabolism-cytochrome P450, metabolism of xenobiotics by cytochrome P450, drug metabolism-other enzymes, steroid hormone biosynthesis, protein digestion and absorption, and fat digestion and absorption (Figure 3B).

### 2.4. qRT-PCR Verification of Flavone-Regulated Genes

**Figure 4:**
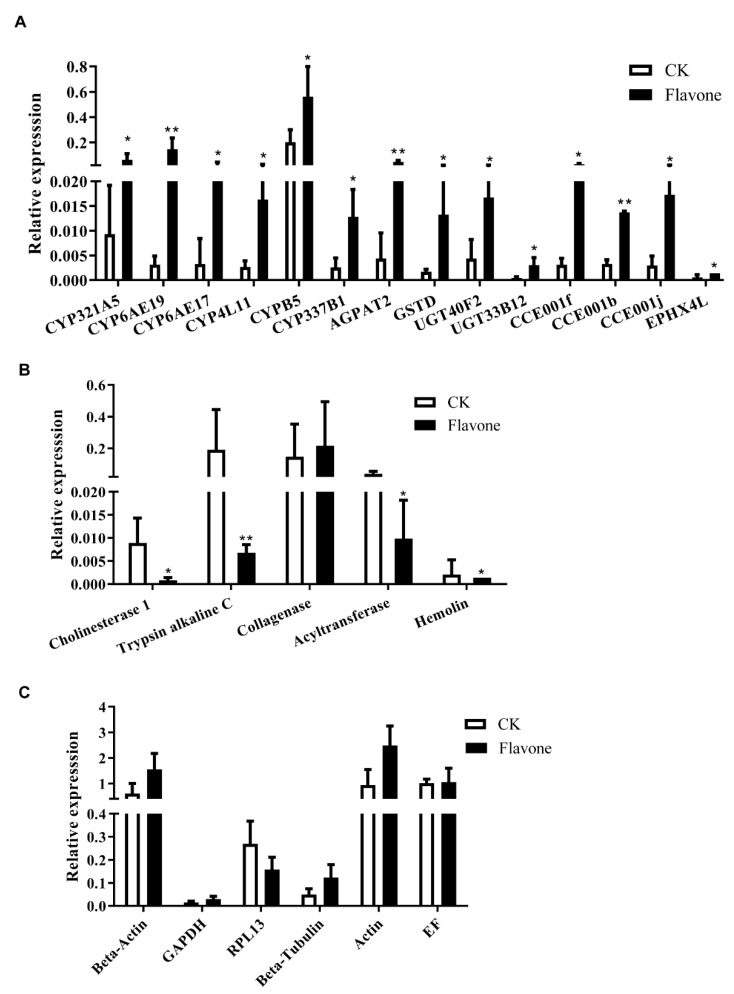
Correlation between the relative expression levels and FPKM values of 14 flavone-upregulated (**A**), 5 flavone-downregulated (**B**), and 6 non-flavone-responsive genes (**C**). (**A**,**B**): One-tailed *t* test at *p* < 0.05. (**C**) Two-tailed *t* test at *p* < 0.05. Bar pairs with one (*p* < 0.05, independent *t* test) and two asterisks (*p* < 0.01, independent *t* test) are significantly and extremely different, respectively.

Fourteen flavone-upregulated genes (six P450s (*CYP6AE19*, *CYP6AE17*, *CYP321A5*, *CYP337B1*, *CYP4L11*, *CYPB5*), four transferases (*UGT33B12*, *UGT40F2*, *GSTD*, *AGPAT2*), and four carboxylesterases (*CCE001f*, *CCE001b*, *CCE001j*, *EPHX4L*)), five flavone-downregulated genes (*1-acyl-sn-glycerol-3-phosphate acyltransferase alpha-like*; *cholinesterase 1-like*; *trypsin*, *alkaline C-like*, *hemolin* and *collagenase-like*), and six non-flavone-responsive genes (*Actin*, *beta-Actin*, *beta-Tubulin*, *Elongation factor 1 alpha* (*EF*), *Glyceraldehyde-3-phosphate dehydrogenase* (*GAPDH*), and *Ribosomal protein L13* (*RPL13*)) were verified by qRT-PCR. qRT-PCR analysis showed that all the 14 flavone-upregulated genes revealed by RNA-seq had significantly higher expression (one-tailed *t* test at *p* < 0.05) in the flavone-treated larvae than in the control larvae (Figure 4A). Four out of the five flavone-downregulated genes were expressed at a significantly lower level (one-tailed *t* test at *p* < 0.05) in the flavone-treated larvae (Figure 4B) and no differences were observed for the six non-flavone-responsive genes between the flavone and control groups (two-tailed *t* test at *p* < 0.05) (Figure 4C). Correlation analysis uncovered that the relative expression levels of these genes obtained by qRT-PCR matched well with their FPKM values derived from RNA-seq (*R*^2^ = 0.8386).

### 2.5. Identification of Conserved Motifs in the Promoter Regions of Flavone-Upregulated Genes

**Figure 5:**
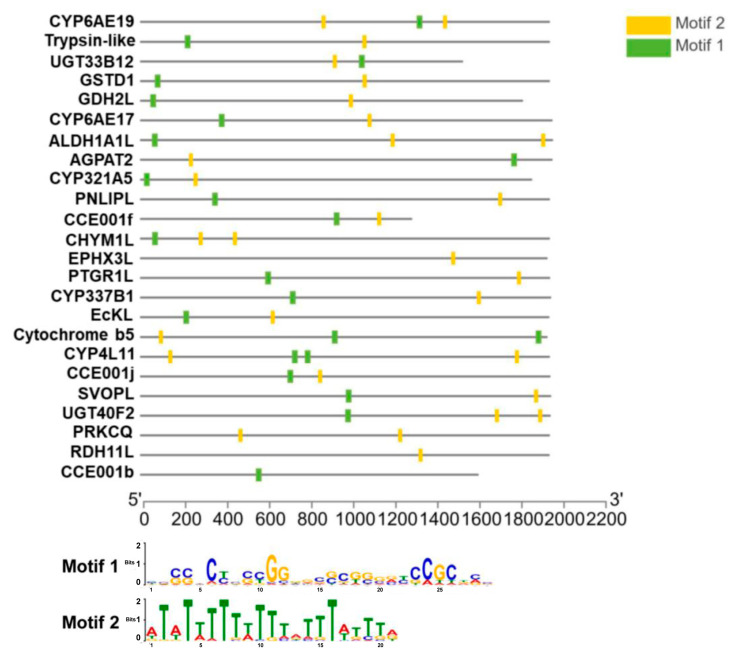
Conserved motifs present in the promoter regions of 24 flavone-upregulated genes revealed by the MEME program, with width set to 6–30.

We used the MEME program to screen the promoter regions (up to 3 kb upstream of the start codon) of 24 flavone-upregulated genes (see their names in Figure 5) for the presence of conserved motifs that may act as xenobiotic response elements to flavone (XRE-Fla). The 24 genes were selected from the 38 flavone-upregulated genes (Figure 2C) using the following two criteria: (1) FPKM value greater than 5; and (2) significantly higher relative expression in the flavone-treated larvae (two-tailed *t* test at *p* < 0.05). One 29 bp GC-rich motif called Motif 1 and one 21 bp TA-rich motif called Motif 2 were detected from the promoter regions of these genes (Figure 5). Motif 1 was present in the promoter regions of 21 of the 24 genes, whereas the promoter regions of all the 24 genes had Motif 2 (Figure 5).

### 2.6. Identification of Known Elements in the Promoter Regions of Flavone-Upregulated Genes

**Figure 6:**
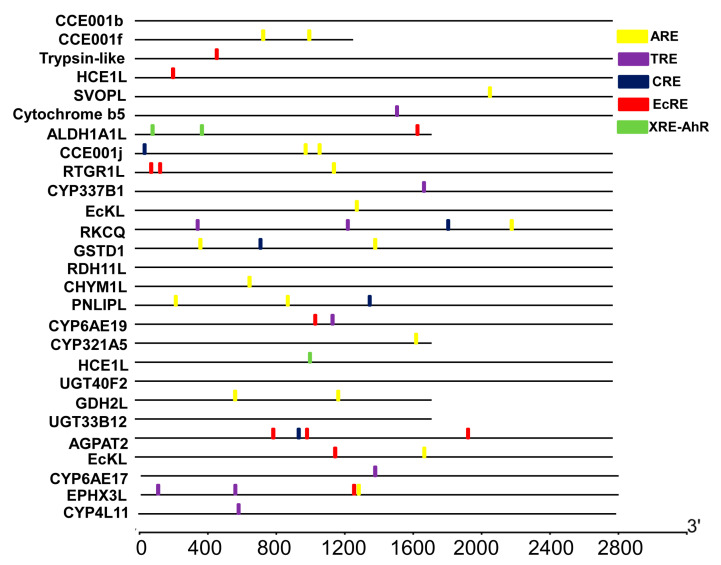
Putative *cis*-acting elements identified in the promoter regions of the upregulated genes. Yellow is the ARE (GCNNNGTCA) element, purple is TRE (TGAC(G)TCA), blue is CRE (TGACGTCA), red is EcRE (G(G/T)T(C/G)ANTG(A/C)(A/C)(C/T)(C/T)), and green is XRE-AHR (T(A/T) GCGTG).

We also screened the promoter regions of the 24 flavone-upregulated genes for the presence of previously characterized *cis*-acting elements including XRE (Xenobiotic response element), TRE (TPA response element), CRE (cAMP-response element), EcRE (Ecdysone receptor element), and XRE-AhR (Xenobiotic response element-Aryl hydrocarbon receptor nuclear) that may mediate the flavone induction of these genes. Among the five known elements, ARE, EcRE, TRE, CRE and XRE-AhR were detected in the promoter regions of 13, 6, 4, 3, and 1 flavone-upregulated genes, respectively (Figure 6). Given its high prevalence, ARE is likely to be one of the elements that respond to flavone.

### 2.7. Functioanl Validation of Motif 1, Motif2 and ARE 

**Figure 7:**
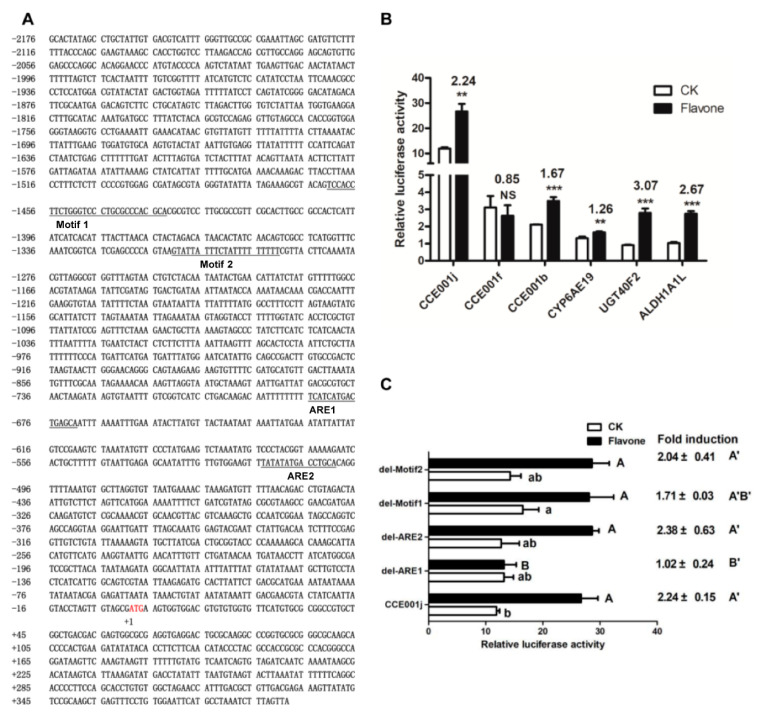
Verification of Motif1, Motif2, and ARE: (**A**) Promoter, 5′ UTR sequence of *CCE001j*. The start codon ATG is numbered with +1 and marked in red, the upstream sequence is preceded by a “−” and the downstream sequence by a “+”. The predicted Motif1, Motif2, ARE1 and ARE2 are underlined. (**B**) The Renilla/reference firefly luciferase ratio of 6 upregulated gene promoters before and after flavone induction. (**C**) The Renilla/reference firefly luciferase ratio of Motif1, Motif2, ARE1, and ARE2 deleted recombinant plasmids was calculated. The structure of the internal deletion is named “del-” and the name of the deletion sequence. Bars with different letters such as a, b (A, B or A’, B’) (*p* < 0.05, one-way ANOVA followed by Tukey’s HSD test) are significantly different. Bar pairs with two and three asterisks mean *p* < 0.01 and *p* < 0.001, respectively. NS means no significantly different (*p* > 0.05, independent *t* test).

We subcloned the promoter regions of six flavone-upregulated genes, namely *CCE001j, CCE001b, CCE001f, CYP6AE19, UGT40F2*, *ALDH1A1L* into, and three carboxyleesterases belonging to CCE clade 001. The promoters of these six genes link to the pGL3 plasmid. The luciferase activity was recorded 48 h after transfection of the recombinant plasmid into *cotton bollworm* adipose cells. After 48 h, the fluorescence values of the control group and the flavones treatment group were detected. *We found that CCE001j* increased from 11.90 ± 0.43 to 26.70 ± 2.41 (*p* = 0.001) *and CCE001f* decreased from 3.11 ± 0.55 to 2.63 ± 0.50 (*p* = 0.41) after flavones induction. *CCE001b* increased from 2.10 ± 0.02 to 3.50 ± 0.18 (*p* = 0.0004) after induction by flavones. *CYP6AE19* increased from 1.32 ± 0.07 to 1.66 ± 0.04 (*p* = 0.005) after induction by flavones. *UGT40F2* increased from 0.91 ± 0.03 to 2.79 ± 0.21 (*p* = 0.0002) after induction by flavones. The flavones of *ALDH1A1L* increased from 1.03 ± 0.05 to 2.75 ± 0.11 (*p* = 0.00003). Among them, *CCE001j* promoter had the highest basal and post-inducible activity among the 6 genes, with basal Renilla/Firely of 11.90 ± 0.43 and inducible Renilla/Firely of 26.70 ± 2.41 and induction multiple of 2.24 (Figure 7B). Therefore, we selected this gene for subsequent element validation (Figure 7A).

Meanwhile, using the pGL3-*CCE001j* promoter as the template, the luciferase activity of four recombinant plasmids obtained by deletion del-Motif1, del-Motif2, del-ARE1, and del-ARE2 was determined. Only the basal activity of del-Motif1 was significantly higher than that of the pGL3-*CCE001j* promoter, which increased from 11.90 ± 0.43 to 16.49 ± 2.30. The activity of del-ARE1 decreased significantly from 26.70 ± 2.41 to 13.22 ± 1.74 (*p* = 0.003). The deletion of the ARE1 element caused the *CCE001j* promoter to be no longer induced by flavones (Figure 7C).

## 3. Discussion

Studying the response of *H. armigera* larvae to flavone will broaden our understanding of the effects of flavone on herbivorous insects and help us lay the foundation for future pest control [31]. In lepidopteron insects, midgut tissue plays an important role in feeding and toxin metabolism, which means it is the main tissue affected by the flavone’s toxin [32]. In this study, transcriptomics were used to analyze gene expression in the midgut of *H. armigera* treated with flavone, and a flavone-induced gene change pattern was obtained. Based on these differentially expressed genes, GO analysis and KEGG analyses were performed. Finally, *cis*-regulatory elements in promoters of upregulated gene expression were predicted and the functions of novel and classic elements were verified in the *CCE001j* promoter.

In this study, three flavone concentration gradients were set, which were 0.01%, 0.1% and 1%. After feeding, the fecal volume of *H. armigera* larvae decreased significantly with the increase in the concentration of flavone, indicating that a high concentration of flavone can inhibit feeding of *H. armigera*. The body weight of larvae treated with 0.01% and 0.1% flavone decreased slightly, but not significantly. After treatment with 1% flavone concentration, body weight decreased significantly. This was consistent with the reported effects of flavone on *Spodoptera litura* [10], indicating that the low flavone concentration did not affect insect body weight in a short period of time, which might be because of the role of detoxification metabolism genes in insects. A 0.1% concentration was commonly used in previous studies of flavone-induced gene expression [30]. Therefore, in this study, 0.1% flavone concentration was selected to treat the 6th larva of *H. armigera*, enabling the larvae to fully mobilize the detoxification gene to metabolize flavone and take midgut tissue for transcriptome sequencing.

RNA-seq revealed a total of 48 differentially expressed genes including 38 upregulated gens and 10 downregulated genes. Among the upregulated genes were P450s, carboxylesterase, UGTs, and glutathione transferase; these genes have been reported to be involved in plant toxin metabolism [31,33,34]. The upregulated DEGs were mainly enriched in drug metabolism-cytochrome P450, metabolism of xenobiotics by cytochrome P450, and drug metabolism-other enzymes. These results indicated that detoxification-related pathways play an important role in the interaction between *H. armigera* and flavone. Only 10 downregulated genes were detected, and future research should also focus on these genes, particularly on these genes’ downregulated mechanisms and their downregulated functions, especially in antifeeding and insect development.

As the FPKM values of some upregulated genes were relatively low, and the expression trends of some genes were inconsistent with the transcriptome results, 24 promoters of upregulated genes were selected for *cis*-regulatory element prediction. Two motifs were predicted by the online site MEME and some *cis*-regulatory elements previously reported to be associated with toxins were also predicted. Six promoters of upregulated genes were cloned. Except for the *CCE001f* promoter, the other five promoters significantly increased luciferin activity after induction by flavones, but *CCE001b*, *CYP6AE19*, *UGT40F2*, and the basic and induced activity of *ALDH1A1L* were low, so the *CCE001j* promoter was selected to predict the function of the element.

Four potential *cis*-acting elements Motif1, Motif2, ARE1 and ARE2 in the *CCE001j* promoter were verified by deletion of the elements. The results indicated that the two motifs obtained by MEME prediction were not induced by flavones, but internal deletion of Motif1 significantly enhanced basal promoter activity of CCE001j, and Motif2 may plays a role in other aspects, which needs further study.

In conclusion, our results suggest that high doses of flavone have an antifeeding effect on *H. armigera*, and *cis*-acting elements play an important role in activating detoxification metabolic pathways in insects. In particular, the ARE element in the *CCE001j* promoter determines whether the gene is induced by flavone. The results of this study will help us to understand the reaction process of flavonoids in *H. armigera* and help us to clarify the action mode of flavone.

## 4. Conclusions

Transcriptome sequencing was performed for the first time in the midgut tissues of *H. armigera* treated with flavone. In terms of a bioassay, we proved that low doses of flavone did not affect the feeding and growth of *H. armigera*, while high doses of flavone hindered larval feeding and caused the slow growth of *H. armigera*. In terms of transcriptome sequencing, we reported for the first time differentially expressed genes in the midgut tissues of *H. armigera* after feeding on flavone, and these genes were mainly enriched in detoxification metabolic pathways. *Cis*-acting elements in the promoter regions of some upregulated genes were predicted. Finally, the response of some components to flavone was verified in the *CCE001j* gene promoter. In addition, it was confirmed that element ARE1 in the *CCE001j* promoter determines whether the promoter is induced by flavone. These results indicated that the expression levels of detoxification metabolism genes in midgut tissues were upregulated after feeding on flavone, and *cis*-acting elements in these genes played an important role in this process, such as the ARE1 element in *CCE001j* promoter, which determined whether the promoter was induced by flavone.

## 5. Materials and Methods

### 5.1. Insects

The *H. armigera* strain used in this study was collected from tobacco fields in Xuchang City (Henan Province, China) in 2018. The larvae were reared on a wheat germ-containing artificial diet [35] at 26 ± 1 °C, 60 ± 10% relative humidity (RH), and a photoperiod of 16:8 h L/D. The larvae were separated into plastic cups individually. Adult moths were placed in plastic mating cages (5 pairs/cage) with 10% honey solution. The cage was covered with a piece of cheese cloth sheet for collecting eggs.

### 5.2. Flavone Feeding Bioassay

Flavone (CAS number: 525-82-6, purity 98%) was purchased from Shanghai Aladdin Biochemical Technology Co. (Shanghai, China). Three different final concentrations (0.01%, 0.1% and 1%, *w*/*w*) of flavone were incorporated into a corn flour/soybean flour-based diet. Briefly, when the diet was cooled to about 47 °C, 400 µL of the corresponding concentration of flavone dissolved in dimethyl sulfoxide (DMSO) was added to 40 mL of diet, vortexed to mix well, and then evenly poured into 27 plastic cups. After the diet was cooled and solidified, *H. armigera* 5th instar larvae of similar size were transferred to the 27 plastic cups (1 larva per cup). Three days later, the body weight and feces of each larva were weighed and counted. There were 3 biological replicates of 9 larvae each per concentration and DMSO control.

### 5.3. RNA Extraction and Library Construction and Illumina RNA-Seq

Newly molted sixth instar of *H. armigera* were individually transferred to cups (1 larva/cup) with control or 0.1% flavone diets. There were 3 biological replicates in both the experimental group (0.1% flavone diets) and the control group (diets with equal volume of the solvent DMSO), with 6 larvae in each replicate. After 48 h of treatment, midgut tissue was dissected, and a quick-frozen tube stored at −80 °C.

Total RNA of the above six larval midgut samples were extracted according to the steps using RNA extraction kit (Beibei Biological, Zhengzhou). RNA purity, integrity and concentration were determined by Nanodrop 2000 spectrophotometer (Wilmington, DE) cDNA libraries, and then sequenced on Illumina HiSeq 2500 platform by Gene Denovo Biotechnology Co. (Guangzhou, China).

### 5.4. DEGs Analysis

The expression levels were displayed as original reads count and FPKM. The original reads count represented the number of reads contained in the transcript, but being affected by sequencing amount and gene length, the original reads count was not conducive to the comparison of differential genes between samples. To ensure the accuracy of the subsequent analysis, we first corrected the sequencing depth and then corrected the length of genes or transcripts. After obtaining the FPKM value of genes, gene differential expression analysis was conducted. The input data for the gene differential expression analysis were reads count data obtained from gene expression level analysis, which was analyzed by DESeq2 software [36]. The analysis was mainly divided into three parts: Read count was first standardized. Then the hypothesis testing probability (*p*-value) was calculated according to the model. Finally, multiple hypothesis testing was performed to obtain the FDR value (error detection rate). Genes with a FDR< 0.05 and | log2FC | > 1 were considered as differentially expressed genes (DEGs).

### 5.5. Expression Validation of DEGs by qRT-PCR

Real-time quantitative fluorescence PCR (qRT-PCR) was used to verify the DEGs identified from sequencing analysis. All primers were designed using Primer 5.0 software (Premier Biosoft International, Palo Alto, CA, USA) and DNAMAN (Lynnon Biosoft, San Ramon, CA, USA) according to strict principles (primers are listed in Table S1). The qRT-PCR reactions were performed following the manufacturer’s manual for the UltraSYBR Mixture (low ROX) (CWBIO, Beijing, China) on an ABI QuantStudio 5 (manufacture information). Each reaction (20 μL final volumes) contained 1.0 μL cDNA, 10 μL UltraSYBR Mixture (low ROX), 0.5 μL forward and reverse primers, and 8 μL ddH_2_O. PCR conditions were as follows: 10 min at 95 °C, followed by 40 cycles of 10 s at 95 °C, 30 s at 60 °C, 32 S at 72 °C and 10 min at 72 °C *EF* and *Actin* (ACT) were used as internal controls. Three biological samples were repeated with three techniques repeats each. The relative expression levels of each gene were calculated by the 2^−ΔΔCT^ method [37]. The 2^−ΔΔCT^ method was used to process the data, and the processed data were plotted by GraphPad Prism software. Primer sequences and amplification efficiency are shown in Table S1.

### 5.6. Conservative Motif Analysis of Flavone-Induced Upregulated Genes in H. armigera

The purpose of MEME (Multiple EM for Motif Elicitation) is to allow users to discover signals (called ‘motifs’) in DNA or protein sequences. The user of MEME inputs a set of sequences believed to share some (unknown) sequence signal(s). For example, some or all of a set of promoters from co-expressed and/or orthologous genes may contain binding sites (the ‘signal’) for the same transcription factor. We used the online version of MEME (Multiple EM for Motif Elicitation) (http://meme-suite.org/tools/meme, accessed on 5 November 2021) to predict transcription factor binding sites in upregulated gene promoters. To put it simply, we started by uploading a FASTA file containing the sequence of 24 upregulated gene promoters into MEME. Finally, we changed some parameters, changed the Motif width parameter from 6 to 30, and set the number of pre-find motifs to 20. At the end of the task, we created MEME.xml and MAST.xml files. We visualized the MEME/MAST xml results with TBtools (bioinformatics analysis software). In addition, ALLGEN and JASPAR software were used to predict known gene toxin-related response elements in promoters.

### 5.7. Construction of Wildtype and Deletion Promoter-pGL3 Constructs

Promoters of six genes (*CCE001j*, *CCE001b*, *CCE001f*, *CYP9AE19*, *UGT40F2*, and *ALDH1A1L*) were amplified from genomic DNA. The Mlu1 restriction site was designed on the forward primers (BglII was designed for the forward primers of *CCE001j*), and the XhoI restriction site was designed for all reverse primers (HindIII was designed for the reverse primers of *CCE001j*). The PCR products were analyzed by electrophoresis on 1.5% agarose gel; the target bands were recovered from the gel and double-digested by Mlu1 and XhoI (BglII and HindIII for *CCE001j*). The recombinant plasmid was obtained by attaching the double-digested products to firefly luciferase reporter vector pGL3-basic (XhoI and MluI double-digested) (double-digested products of *CCE001j* were attached to BglII and HindIII digested pGL3-basic).

The 4 internal constructs of *CCE001j* were made by PCR using the reverse primers CCE001j-del-Motif1-R, CCE001j-del-Motif2-R, CCE001j-del-ARE1-R, CCE001j-del-ARE2-R and the forward primers CCE001j-del-Motif1-F, CCE001j-del-Motif2-F, CCE001j-del-ARE1-F, and CCE001j-del-ARE2-F (see supplementary Table S2 for their sequences). Each PCR amplification was performed in a 50 μL mixture containing 25 μL Prime Star, 2 μL forward primer, 2 μL reverse primer, 1 μL template, and 20 μL ddH2O. The PCR conditions were as follows: 3 min initial denaturation at 98 °C, followed by 30 cycles of 10 s denaturation at 98 °C, 10 s annealing at 58 °C, 4 min extension at 72 °C, and a 5 min final extension at 72 °C.

### 5.8. Transient Transfection and Dual Luciferase Assay

One hundred uL of *H. zea* fatbody cells were seeded onto wells of 96-well plates (5 × 10^5^ cells/well). After 30 min, the seeded cells were transiently co-transfected with either of the 6 wildtype (pGL3-CCE001j promoter, pGL3-CCE001b promoter, pGL3-CCE001f promoter, pGL3-CYP6AE19 promoter, pGL3-UGT40F2 promoter, pGL3-ALDH1A1L promoter) and 4 deletion promoter-pGL3 constructs (del-Motif 1, del-Motif 2, del-ARE1 and del-ARE2) (0.1 µg/well) and the internal renilla luciferase control reporter plasmid PHRL-TK (Promega, 0.01 µg/well). There were three technical replicates in both the experimental group and the control group. Six hours after transfection, the final concentration of 18.5 μM flavone (induced) or equivalent volume of methanol (control) was added. After 48 h, the cells were harvested and the luciferase activity of kidney and firefly was measured using the dual luciferase reporting assay system (Promega, Madison, WI, USA) on a TD-20/20 single-tube luminescence instrument designed by Turner (Turner Biosystems, Sunnyvale, CA, USA). The relative firefly luciferase activity normalized against the renilla luciferase activity was calculated as an indicator of the basal or flavone-inducible promoter activity of each construct. At least 3 repeated measurements were conducted for each independent transfection. 

## Figures and Tables

**Table 1:** Sequencing data of the larval midgut RNA samples from the 48 h old control and flavone-treated 6th instar larvae of *H. armigera*.

Sample	Raw Reads	Clean Reads	GC%	Q20 (%)	Q30 (%)	Total Mapped	Mapped Ratio (%)
CK-1	37,507,152	37,459,394	55.9	97.85	93.94	26,289,514	74.44
CK-2	60,200,136	60,101,208	55.3	97.96	94.36	42,996,004	74.97
CK-3	52,278,332	52,184,106	55.0	97.81	94.02	33,662,201	70.24
Fla-1	58,684,188	58,567,088	55.4	97.66	93.68	40,176,673	73.15
Fla-2	48,074,836	47,994,546	56.1	97.55	93.40	33,086,674	73.19
Fla-3	37,538,966	37,504,354	55.1	97.98	94.06	26,365,336	73.74

## Data Availability

RNA sequences were submitted to the SRA (Sequence Read Archive) database of the NCBI with accession SRP407813.

## Supplementary Materials

The following supporting information can be downloaded at: https://www.mdpi.com/article/10.3390/toxins15060365/s1, Table S1: qRT-PCR primers for 6 unchanged genes and 18 differentially expressed genes; Table S2: Primer sequences used for construction of promoter-pGL3 constructs.
