# Supplementary material for: Identification of the Flavone-Inducible Counter-Defense Genes and Their cis-Elements in Helicoverpa armigera
Source: Toxins (Basel). 2023 May 29;15(6):365. doi: 10.3390/toxins15060365 (PMC10303759; doi:10.3390/toxins15060365)
Supplement: Supplementary file 1 [file toxins-15-00365-s001.zip › toxins-2372285-supplementary.pdf]

toxins

MDPI

Table S1. qRT-PCR primers for 6 unchanged genes and 18 differentially expressed genes.

Gene	Orientation	Primer sequences (5′-3′)	Product length (bp)	Primer amplification efficiency (%)
CYP321A5	FORWARD	CAAGAACAAAGTAATTGGACCATCT	197	97.8
	REVERSE	GATTGAAAGATTGAAAGTCTGAGGA		
CYP6AE19	FORWARD	AGCAAGAAAATTACAATGAAGGTTG	199	91.2
	REVERSE	CGTAGTTGATTTTGTTGTTATGACG		
CYP6AE17	FORWARD	CTTTAGTGTGGGTTTAAGGGTTTTT	200	103.2
	REVERSE	GCTGTTGTACAGCTTATTGAAGACA		
CYP4L11	FORWARD	AACTATATTTGGCAGTGAAATGGAA	200	100.1
	REVERSE	TCTGATAGATGTTGATGAGGACTGA		
cytochrome b5	FORWARD	AGGAGCTCATGAAGAAGTACGTG	236	110.2
	REVERSE	AGTCTGTAATGGTTGCATTGTGTC		
CYP337B1	FORWARD	TTTACGACCATCGTTTATTCAAAAT	201	93.4
	REVERSE	TTCTTCTGTAGTTCCAAGCAAATCT		
CCE001f	FORWARD	ATTGACTTGTTTGAAGATTGGAAAG	198	107.6
	REVERSE	AACAAACAGAACATCCGAAAAGTAG		
CCE001b	FORWARD	GTGAGCTTTTAAAATCGACACAGTT	206	95.0
	REVERSE	AATAGTAACATCATCAGGGTTACCG		
CCE001j	FORWARD	TGGAACTGTTAACCTCAGATCATTT	203	95.6
	REVERSE	GTCATATATCATAACGCGAACCAAT		
EPHX3L	FORWARD	CAGAGGAAAGCATCTTGGTATGTAT	199	99.1
	REVERSE	GGTAGTAGTTTATAGGTGGCGTGAG		
AGPAT2	FORWARD	ATCTTTACGCGCTAGCAATTATATG	193	107.6
	REVERSE	TTTCGCTTGTGATTCTTAATAGCAT		
GSTD	FORWARD	AAAGATCCCAGATGTAAAGTACGTG	203	102.8
	REVERSE	TCCTTAGATCAGTTGGGTAAAGAGA		
UGT40F2	FORWARD	AAATAAGAATCCCCATCCAAAATTA	200	89.6
	REVERSE	GATCCTTCAACATTTTCTGTACGTT		
UGT33B12	FORWARD	TGCTGTTTTTGAATATCCATCCTAT	204	106.3
	REVERSE	GTACCTTCTCTGGAGGTAACAGTGA		
Actin	FORWARD	GACGGTCAGGTCATCACCATC	151	111.7
	REVERSE	ACAGGTCCTTACGGATGTCA		
Beta-actin	FORWARD	CCTGGTATTGCTGACCGTATGC	147	109.7
	REVERSE	CTGTTGGAAGGTGGAGAGGGAA		
Beta-tubulin	FORWARD	AGCAGTTCACCGCTATGTTC	106	122
	REVERSE	AGGTCGTTCATGTTGCTCTC		
GAPDH	FORWARD	CCAGAAGACAGTGGATGGAC	140	127
	REVERSE	TACCAGTCAGCTTTCCGTTC		
EF-1 alpha	FORWARD	GAAGTCAAGTCCGTGGAGATG	171	107.9
	REVERSE	GACCTGTGCTGTGAAGTCG		
RPL13	FORWARD	CTGCAAGACGTCACCGCAG	139	110
	REVERSE	CCACGACCAGCACGAACCT		
Cholinesterase 1-like	FORWARD	AAAGGGAAGAAGTGGCGAAT	208	122
	REVERSE	ACAACCGGAGTTTCATCGTC		
Trypsin alkaline C-like	FORWARD	ACTTCGCTATCATGCGCTCT	224	120
	REVERSE	AACATATCTGCCGGCGTAAC		
Collagenase-like	FORWARD	TGGTGGTAGTGTCAGCCAAA	184	122

	REVERSE	GCGTCTGTTGTTGCTGGTTA		
1-acyl-sn-glycerol-3-phosphate acyltransferase alpha-like	FORWARD	GAGCCTACAAACAATTGAAAGTCAC	192	101
	REVERSE	TTATGAAATAGTAGGGCGAGAACAC		

Table S2. primer sequencesused for construction of promoter-pGL3 constructs.

**Oligonucleotide**	**Orientation**	**Primer sequences (5′-3′)**
CCE001j	FORWARD	GCACGACGCGTACACTGGTGGAGGGTAGGGTGC
	REVERSE	GACCGCTCGAG TCTCGTCAACAGCGTCAAATGG
CCE001b	FORWARD	GCACGACGCGTGTGAGCGATTGACCCCATTCGAGGCGATC
	REVERSE	GACCGCTCGAGGTGCGTTAATTACGTTACATGTTGTAT
CCE001f	FORWARD	GCACGACGCGTCTGATTATCTTAGAAACAACGAGGGT
	REVERSE	GACCGCTCGAG CTGTTCGTACTTGTGGCGGTAG
CYP9AE19	FORWARD	GCACGACGCGTGTTACGCGGAAGCGTAGAAGTTTGCTG
	REVERSE	GACCGCTCGAGAGACGTGAAGAGTGGAGTCAGGTTCTG
ALDH1A1L	FORWARD	GCACGACGCGTCACTCTGAACGTCTATTCGCATCAATCG
	REVERSE	GACCGCTCGAGAACAGCCCTGAATCAGTAACCACCATAA
UGT40F2	FORWARD	GCACGACGCGTCTACCTTCTAAAATCTGACTGCCATTTACAT
	REVERSE	GACCGCTCGAGACATTAAGATCTAAAATTACTTACTTCATGG
CCE001j-del-motif 1	FORWARD	AGAAAGCGTACAGCGCGTCCTTGCGCCGTTCGCACTTGCCGCCACTCA
	REVERSE	CGATAGCGTAGGGTATATTATAGAAAGCGTACAGCGCGTCCTTGCG
CCE001j-del-motif 2	FORWARD	CGAGCCCCAGTAACGTTACTTCAAAATACGTTAGGCGTGGTTTAGTAA
	REVERSE	TCATGGTTTCAAATCGGTCATCGAGCCCCAGTAACGTTACTTCAAA
CCE001j-del-ARE1	FORWARD	ATTTAAAATTTGAAATACTTATGTTACTAATAATAAATTATGAAATATTATTA TGTCCG
	REVERSE	AAAAAAAATTGTCTTGTCAGGATGACCGACAAATTACACTTATCTTAGTT
CCE001j-del-ARE2	FORWARD	ATCTTTACGCGCTAGCAATTATATG
	REVERSE	TTTCGCTTGTGATTCTTAATAGCAT
